# Comparative Analysis of Sepsis Outcomes Across Body Mass Index Groups: A Retrospective Cohort Study

**DOI:** 10.3390/jcm14238501

**Published:** 2025-11-30

**Authors:** Abdulmajeed M. Alshehri, Lama Alfehaid, Saad Alhamdan, Mohammad S. Shawaqfeh

**Affiliations:** 1 Department of Pharmacy Practice, College of Pharmacy, King Saud bin Abdulaziz University for Health Sciences, Riyadh 11481, Saudi Arabia; 2 King Abdulaziz Medical City, National Guard Health Affairs, Riyadh 14611, Saudi Arabia; 3 King Abdullah International Medical Research Center, Riyadh 11481, Saudi Arabia

**Keywords:** BMI, sepsis, septic shock, mortality

## Abstract

**Background/Objectives**: The relationship between body mass index (BMI) and clinical outcomes in sepsis patients remains controversial, with some studies suggesting an “obesity paradox” and others indicating increased risks for underweight individuals. This study aims to further explore the impact of BMI on mortality and other specific sepsis outcomes. **Methods**: This was a retrospective cohort study of adult patients with sepsis admitted to the intensive care unit (ICU) from 1 January 2021 to 31 December 2023. Patients were divided into four groups according to BMI category. The primary outcome of this study was ICU mortality. Secondary outcomes included the development of septic shock, acute respiratory distress syndrome, 30- and 90-day mortality, ICU and hospital length of stay, and vasopressor- and/or ventilation-free days. **Results:** A total of 559 patients were included in the study. Among these, 51 were in the underweight group, 206 were in the normal weight group, 158 were in the overweight group, and 184 were in the obese group. The primary outcome of ICU mortality was not significantly different among all BMI groups (*p*-value > 0.05). Similarly, all secondary outcomes were not significantly different between the groups. **Conclusions**: Our findings demonstrate that BMI in sepsis patients is not associated with worse clinical outcomes. Further prospective research is warranted to confirm these findings on a larger scale.

## 1. Introduction

Sepsis, a life-threatening organ dysfunction caused by a dysregulated host response to infection, remains a significant challenge in intensive care units (ICUs) worldwide [1]. It is a leading cause of mortality and morbidity, particularly among critically ill patients [2]. The complexity of sepsis arises from its multifactorial nature, involving a cascade of inflammatory responses that can lead to septic shock, acute respiratory distress syndrome (ARDS), and multiple organ failure [1,3]. Despite advances in medical care, managing sepsis remains challenging and requires further research to identify factors affecting patient outcomes.

Body mass index (BMI), a measure of body fat based on height and weight, is a widely used indicator to classify individuals into categories such as underweight, normal weight, overweight, and obese [4]. BMI has been increasingly recognized as an important factor influencing sepsis outcomes due to its physiological effects on inflammatory response, pharmacokinetics, and metabolic reserve [4]. The relationship between BMI and clinical outcomes in sepsis patients has been a subject of considerable debate. Some studies suggest that obesity may confer a protective effect, a phenomenon often referred to as the “obesity paradox,” where overweight and obese individuals exhibit lower mortality rates compared to their normal-weight counterparts [5,6,7]. Conversely, other research indicates that underweight patients may experience higher mortality rates, potentially due to malnutrition and decreased physiological reserves [8]. A study involving mechanically ventilated patients with lung injury revealed that being underweight (BMI < 18.5 kg/m^2^) heightened the risk of death, whereas a higher weight up to a BMI of 40 kg/m^2^ decreased this risk [9]. Furthermore, low muscle mass has been shown to be correlated with longer ICU stays, increased duration of mechanical ventilation, and a greater risk of mortality, as it can contribute to respiratory muscle weakness [10]. These inconsistencies highlight the need for well-designed cohort studies evaluating BMI subgroups in real-world settings.

This study aimed to further examine the relationship between BMI and specific clinical outcomes related to sepsis. These outcomes include ICU mortality, in-hospital mortality, the length of stay (LOS) in both the hospital and ICU, progression to septic shock, development of acute respiratory distress syndrome (ARDS), and the number of ventilation-free days and vasopressor-free days within 30 days.

## 2. Materials and Methods

### 2.1. Patients and Study Design

**Figure 1:**
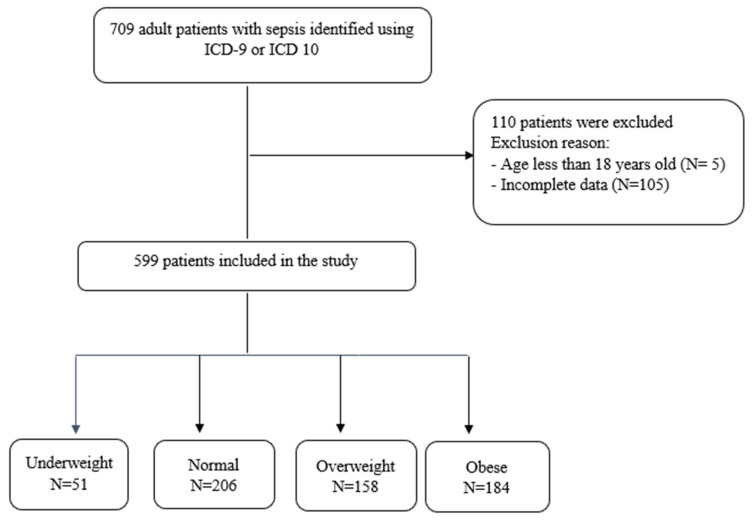
Study flowchart.

This was a retrospective cohort study of adult patients with sepsis admitted to the ICU at the National Guard Health Affairs (NGHA) hospital in Riyadh, Saudi Arabia, from 1 January 2021 to 31 December 2023. System-generated reports using ICD-9 or ICD-10 were used to extract patients with sepsis. All identified patients were assessed for the inclusion criteria. Patients were included if they were at least 18 years of age and met the sepsis-3 definition of sepsis (having an infection or suspected infection with an increase in Sequential Organ Failure Assessment (SOFA) score ≥2) [1]. Patients were divided into four groups according to their BMI according to the World Health Organization (WHO) criteria: underweight (BMI < 18.5 kg/m^2^), normal weight (BMI 18.5–24.9 kg/m^2^), overweight (BMI 25.0–29.9 kg/m^2^), and obese (BMI ≥ 30.0 kg/m^2^). Patients were excluded if they were <18 years old or if they had missing data (Figure 1).

### 2.2. Study Variables, Data Collection, and Outcomes

The primary outcome of this study was ICU mortality, while the secondary outcomes were in-hospital mortality, hospital and ICU length of stay (LOS), progression to septic shock (defined as vasopressor initiation to maintain mean arterial pressure (MAP) of 65 mm Hg or greater with a serum lactate level greater than 2 mmol/L after at least 30 mL/kg of crystalloid fluid resuscitation), development of acute respiratory distress syndrome (ARDS) (according to the Berlin definition of ARDS), 30-day ventilation-free days (VFD) (defined as the number of days the patient is alive and does not require invasive mechanical ventilation (MV) in the first 30 days of initiating MV), and 30-day vasopressor-free days (defined as the number of days the patient is alive and does not require invasive vasopressors (norepinephrine, epinephrine, vasopressin, dopamine, phenylephrine) in the first 30 days of initiating a vasopressor). Demographic and clinical data, including age, gender, body mass index, comorbidities, vital signs, SOFA score, Glasgow Coma Score (GCS), source of infection, and laboratory parameters—uric acid (µmol/L); lactate (mmol/L); platelets (×10^9^/L); serum creatinine (μmol/L); blood urea nitrogen (mmole/L); weight blood cells (cells/µL); and procalcitonin (µg/L)—were collected.

### 2.3. Data Analysis

To ensure reproducibility, we explicitly documented all steps of data extraction and analysis. Data extraction was performed by two independent reviewers. Continuous variables were described with means and standard deviation and analyzed using the ANOVA test, while categorical data were described with frequencies and percentages and analyzed using the Chi-square test or Fisher’s exact test. All variables with a *p* value < 0.05 were considered to have a significant impact on the outcomes. All data were analyzed using Stata/SE statistical software Version 15.1 (StataCorp LLC, College Station, TX, USA).

## 3. Results

### 3.1. Characteristics of Study Subjects

A total of 709 adult patients with sepsis admitted to the ICU at NGHA Hospital in Riyadh were identified in the screening process. All identified patients were assessed based on the inclusion criteria. Of those, 207 patients were excluded. A total of 599 patients were included in the study. Among these, 51 were in the underweight group, 206 were in the normal weight group, 158 were in the overweight group, and 184 were in the obese group (Figure 1). The summary of demographic and clinical characteristics is presented in Table 1. Baseline characteristics were comparable between all groups except for BMI and gender (*p*-value < 0.0001). Comorbidities in the study population were similar between all groups except for asthma, DM, and liver disease. The prevalence of asthma differed significantly across BMI categories, with the highest rate observed among obese participants (12.0%), followed by normal-weight (7.3%), underweight (3.9%), and overweight (3.2%) individuals (*p* = 0.013). The prevalence of diabetes mellitus also varied significantly across BMI groups, being highest among normal-weight participants (35.9%), followed by obese (27.7%), overweight (24.1%), and underweight (21.6%) individuals (*p* = 0.042).

Similarly, the prevalence of liver disease demonstrated a statistically significant difference among BMI categories, with rates of 11.7% in normal weight, 10.8% in overweight, 9.8% in underweight, and 7.1% in obese participants (*p* < 0.00001). Sources of infection were similarly distributed between all groups, with urinary tract infections being the most common source. Skin and soft tissue infection was statistically significant between all groups (*p*-value < 0.05). The mean laboratory findings were similar between the groups, and there were no statistically significant differences in any laboratory value among all groups (Table 1).

### 3.2. Main Results

As shown in Table 2, there were no statistically significant differences in primary or secondary outcomes across BMI categories. The primary outcome of ICU mortality was comparable among underweight (19.6%), normal-weight (24.3%), overweight (23.4%), and obese (21.7%) groups (*p* = 0.873). In addition to the primary outcome, we analyzed secondary outcomes including ICU length of stay, need for vasopressors, mechanical ventilation duration, and in-hospital complications. Complete results for all BMI categories are presented in Table 2 to ensure transparency and reproducibility. Regarding secondary outcomes, the incidence of septic shock, ARDS, hospital mortality, vasopressor use, and acute kidney injury did not differ significantly across BMI categories (*p* > 0.05 for all comparisons). The proportion of patients who developed septic shock ranged from 54.4% to 72.5%, while ARDS occurred in approximately 61–69% of patients across groups. Similarly, hospital mortality rates were consistent, ranging between 25.7% and 31.4%.

The use of organ support interventions, including vasopressors, renal replacement therapy, and mechanical ventilation was also similar across BMI categories. The percentage of patients initiated on vasopressors ranged from 53.2% to 58.2%, the percentage started on renal replacement therapy ranged from 29.4% to 40.3%, and the percentage requiring mechanical ventilation ranged from 36.1% to 43.1%.

No significant differences were observed in 30-day or 90-day mortality rates (*p* = 0.9578 and *p* = 0.8228, respectively). Similarly, vasopressor-free days, duration of mechanical ventilation, ventilation-free days at 30 days, ICU length of stay (LOS), and hospital LOS did not differ significantly between BMI groups. Overall, BMI category was not associated with differences in ICU or hospital outcomes, including mortality, organ dysfunction, or resource utilization.

## 4. Discussion

Sepsis can be described as an inflammatory response syndrome that is caused by systemic infection and known as a leading cause for mortality. Obesity is a chronic problem that is best described by accumulation of fat and adipose tissues. The interaction between sepsis and obesity has been an outstanding issue for investigation [11]. In this retrospective study with 599 patients, we investigated whether there is any correlation between BMI and clinical outcomes in sepsis patients. A few studies have suggested that obesity may have an influence on the risk of death in critically ill patients with septic shock. A large multicenter retrospective study that included 8670 sepsis patients from several countries showed that obese patients have a lower hospital mortality rate compared to normal-weight patients (OR 0.8, 95% CI 0.62–1.02) [12]. In our study, the hospital mortality was not significantly different across all weight groups. In addition, all other intensive care clinical outcomes related to sepsis were also similar between the groups. These include the development of septic shock, ARDS, or acute kidney injury. The initiation of either vasopressors, renal replacement, or mechanical ventilation was also statistically insignificant across all groups. Similarly to the main outcome of ICU mortality, 30-day and 90-day mortality were also not statistically different between the groups. Finally, the vasopressor- and mechanical ventilation-free days, as well as hospital and ICU stay, were comparable across the groups. This assures that BMI category is not likely to be a prognostic factor in sepsis patients. Interestingly, the prevalence of diabetes mellitus was the highest among the group with normal BMI, which contrasts with the generally established association between obesity and the risk of diabetes. The finding could indicate that weight loss due to illness or chronic disease has taken place among patients with diabetes, thus resulting in lower BMI measured upon admission. Since BMI was measured during hospitalization, it might not accurately represent the pre-morbidity weight status of the patients. A similar mechanism may explain the distribution of liver disease seen across different categories of BMI since metabolic effects of chronic liver dysfunction can result in reduced body weight despite the presence of basic metabolic comorbidities.

Several studies have investigated the correlation between BMI and different clinical outcomes in sepsis patients admitted to the ICU with conflicting results. Only a few studies published have reported increased mortality among obese [13,14,15]. On the other hand, several studies have shown that being underweight or having a low BMI may increase the risk of mortality [16,17,18,19,20]. A recent meta-analysis that combined results from 15 studies (105,159 patients) indicated that overweight and obese BMIs were associated with lower mortality (OR: 0.79, 95% CI 0.70–0.88 and OR: 0.74, 95% CI 0.67–0.82, respectively) [21]. However, there were concerns regarding the validity of this association, such as reliance on BMI as the sole measure of obesity, which might be inaccurate in critically ill patients who often receive fluid resuscitation prior to ICU admission, and lack of considerations of frailty in the study population. Furthermore, publication bias was another concern, as studies with positive or protective BMI effects were more likely to be published. Furthermore, these studies are retrospective studies that lead to clinical heterogeneity in outcome measures, severity of disease, control of confounders, and issues with BMI as the sole indicator of obesity.

The heterogeneity observed across studies evaluating the relationship between BMI and mortality in sepsis can be attributed to several factors, including heterogeneity in BMI categorization, variations in patient populations (such as age, sex, comorbidities, and illness severity), differences in follow-up periods, dereferences in sepsis care delivery, and underlying biological or causal complexities such as malnutrition, muscle mass, and frailty. Evidence has shown that patients with low BMI have a higher mortality rate due to malnutrition or sarcopenia, whereas the obesity paradox may be explained by greater metabolic reserves or altered inflammatory responses [21,22]. In contrast, our study did not detect significant differences in mortality across BMI categories, which may be attributable to the relatively small sample size and the limitations inherent to a single-center cohort. Independent of BMI, modifiable factors such as delay in sepsis diagnosis, delay in antibiotic administration, suboptimal antibiotic dosing, or inappropriate source control remain significant determinants of outcomes in sepsis patients [3]. Therefore, addressing these modifiable risks can enhance our understanding of the impact of BMI on mortality and improve outcomes in patients with sepsis.

There are several limitations of this study that should be acknowledged. First, it has a relatively small sample size, which could have limited its power to detect differences across BMI groups; however, the reasonably convenient sample size with similar baseline characteristics minimizes this limitation. Second, the retrospective study is subject to some unmeasured confounders such as comorbid diseases or unknown pre-admission risk factors that might affect prognosis. Furthermore, mortality as an outcome is multifactorial and could not be attributed to the few known factors. Additionally, this study did not assess nutritional status and muscle mass of the patients, which could impact the outcomes in sepsis patients. Finally, while this study focuses on any relationship between sepsis clinical outcomes and BMI, it does not provide details regarding bacterial cultures, infection management, and ICU protocols. Future studies should include larger, multicenter cohorts with appropriate assessments of nutritional status and muscle mass to better understand the relationship between BMI and sepsis outcomes in order to guide optimized interventions.

## 5. Conclusions

Our findings demonstrate that BMI in sepsis patients is not associated with worse clinical outcomes, including ICU and hospital mortality, development of septic shock, vasopressor use, mechanical ventilation need and duration, and ICU and hospital length of stay. These findings suggest that BMI may not serve as a clinically useful prognostic indicator in sepsis patients. Further prospective research is warranted to confirm these findings.

## Figures and Tables

**Table 1:** Baseline characteristics.

Characteristics	Underweight (*n* = 51)	Normal (*n* = 206)	Overweight (*n* = 158)	Obese (*n* = 184)	*p*-Value
Age (mean ± SD) (years)	65.8 ± 19.7	69.2 ± 17.2	68.3 ± 16.7	68.0 ± 15.5	0.604
BMI (mean ± SD)	16.6 ± 1.93	21.61 ± 1.8	27.5 ± 1.5	37.5 ± 8.9	<0.00001
Male, n (%)	38 (74.5%)	121 (58.7%)	74 (46.8%)	67 (36.4%)	<0.00001
SOFA score (mean ± SD)	6.8 ± 3.6	7.2 ± 3.9	6.7 ± 3.9	6.9 ± 4.1	0.6171
GCS (mean ± SD)	11.8 ± 3.8	12.2 ± 3.5	12.2 ± 3.5	12.6 ± 3.4	0.4215
MAP (mean ± SD)	75.4 ± 20.5	73.6 ± 22.8	78.7 ± 23.7	77.0 ± 25.3	0.2048
Heart rate (mean ± SD)	103.2 ± 30.4	97.8 ± 25.6	99.7 ± 25.9	97.2 ± 26.1	0.4524
Respiratory rate (mean ± SD)	25 ± 6.9	23.4 ± 7.0	23.3 ± 6.7	23.3 ± 6.5	0.4306
Comorbidities					
COPD, n (%)	0 (0)	7 (3.4)	3 (1.9)	10 (5.4)	0.359 (U,N) 1.000 (U,OV) 0.124 (U,OB) 0.524 (N,OV) 0.457 (N,OB) 0.098 (OV,OB)
Asthma, n (%)	2 (3.9)	15 (7.3)	5 (3.2)	22 (12.0)	0.0136
Cancer, n (%)	4 (7.8)	24 (11.7)	17 (10.8)	23 (12.5)	0.8162
Transplant, n (%)	0 (0)	14 (6.8)	5 (3.2)	5 (2.7)	0.0791 (U,N) 0.3394 (U,OV) 0.5881 (U,OB) 0.2516 (N,OV) 0.2561 (N,OB) 0.7603 (OV,OB)
Diabetes mellitus, n (%)	11 (21.6)	74 (35.9)	38 (24.1)	51 (27.7)	0.0424
Liver disease, n (%)	5 (9.8)	24 (11.7)	17 (10.8)	13 (7.06)	<0.00001
Coronary artery disease, n (%)	3 (5.9)	5 (2.4)	5 (3.2)	5 (2.7)	0.6257
CKD, n (%)	17 (33.3)	95 (46.1)	55 (34.8)	72 (39.1)	0.1121
Gout, n (%)	1 (2.0)	3 (1.5)	1 (0.63)	2 (1.1)	0.8428
Source of infection					
Respiratory, n (%)	4 (7.8)	30 (14.6)	17 (10.8)	28 (15.2)	0.3781
Urinary, n (%)	16 (31.4)	43 (20.9)	34 (21.5)	47 (25.5)	0.3438
Abdominal, n (%)	0 (0)	5 (2.4)	5 (3.2)	6 (3.3)	ND
Skin and soft tissue, n (%)	1 (2.0)	10 (4.9)	15 (9.5)	6 (3.3)	0.0315
Bacteremia, n (%)	15 (29.4)	40 (19.4)	44 (27.9)	37 (20.1)	0.1292
Other, n (%)	6 (11.8)	29 (14.1)	13 (8.2)	20 (10.9)	0.3758
Unknown n, (%)	9 (17.7)	49 (23.8)	30 (18.9)	39 (21.7)	0.6429
Laboratory at admission					
Uric acid (umol/L)	385.4 ± 200.0	395.7 ± 202.2	397.1 ± 188.2	443.1 ± 223.3	0.0688
Lactate (mmol/L)	4.81 ± 4.3	3.61 ± 3.5	3.75 ± 6.2	4.27 ± 15.7	0.7012
Potassium (mmol/L)	4.73 ± 1.14	4.60 ± 0.88	4.53 ± 0.88	4.61 ± 0.99	0.4749
Serum albumin	29.3 ± 6.3	30.7 ± 6.8	30.2 ± 7.5	32.0 ± 9.7	0.0769
Creatinine (umol/L)	206.4 ± 195.0	252.2 ± 202.2	215.6 ± 208.0	239.6 ± 220.7	0.2846
CrCl (mL/minute)	57.4 ± 52.8	40.3 ± 41.1	48.8 ± 37.6	46.4 ± 46.4	0.0524
BUN (mmol/L)	17.6 ± 12.9	16.4 ± 18.0	13.8 ± 10.7	14.7 ± 11.6	0.1860
WBC (cells/µL)	13.3 ± 9.3	12.9 ± 8.9	12.4 ± 8.6	13.2 ± 8.8	0.8219
Platelet (×10^9^/L)	271.1 ± 167.4	252.9 ± 160.9	259.6 ± 135.7	259.9 ± 155.1	0.0832
Procalcitonin (µg/L)	15.8 ± 35.3	6.7 ± 187	12.6 ± 55.5	8.2 ± 39.5	0.3072

Abbreviations: U, underweight; N, normal weight; OV, overweight; OB, obese; BMI, body mass index; SOFA, Sequential Organ Failure Assessment; GCS, Glasgow Coma Scale; MAP, mean arterial pressure; COPD, chronic obstructive pulmonary disease; CKD, chronic kidney disease; CrCl, creatinine clearance; BUN, blood urea nitrogen; WBC, white blood cell count; ND, not determined. ANOVA, Chi-square test, and Fisher’s exact test.

**Table 2:** Primary and secondary outcomes.

Outcome	Underweight (*n* = 51)	Normal (*n* = 206)	Overweight (*n* = 158)	Obese (*n* = 184)	*p*-Value
Primary outcome					
ICU mortality, n (%)	10 (19.6)	50 (24.3)	37 (23.4)	40 (21.7)	0.8738
Secondary outcomes					
Development of septic shock, n (%)	37 (72.5)	121 (58.7)	86 (54.4)	110 (59.8)	0.1519
Development of ARDS, n (%)	35 (68.6)	133 (64.6)	102 (64.6)	112 (60.9)	0.7317
Hospital mortality, n (%)	16 (31.4)	53 (25.7)	42 (26.6)	51 (27.7)	0.8674
Started on vasopressors, n (%)	29 (56.9)	116 (56.3)	84 (53.2)	107 (58.2)	0.8295
Development of AKI, n (%)	17 (33.3)	67 (32.7)	56 (35.4)	54 (29.3)	0.6876
Started on renal replacement, n (%)	15 (29.4)	83 (40.3)	49 (31.0)	64 (34.8)	0.2253
Started on mechanical ventilation	22 (43.1)	80 (38.8)	57 (36.1)	74 (40.2)	0.7891
30-day mortality, n (%)	13 (25.5)	52 (25.2)	40 (25.3)	50 (27.3)	0.9578
90-day mortality, n (%)	11 (21.6)	35 (17.0)	25 (15.8)	31 (16.9)	0.8228
Vasopressor-free days (days)	39.7 ± 78.3	33.7 ± 52.3	36.2 ± 67.1	11.9 ± 13.5	0.2040
Duration of mechanical ventilation (hours)	303 ± 301.4	158.4 ± 120.7	132 ± 190.0	240 ± 171.1	0.4861
30-day ventilation-free days	16.7 ± 11.4	18.9 ± 8.9	12.9 ± 11.9	10.5 ± 10.3	0.0891
ICU LOS (hours)	471.3 ± 634.0	340.7 ± 392.3	494.4 ± 522.5	270.7 ± 283.6	0.1962
Hospital LOS (hours)	720.3 ± 1026	798.6 ± 1110	724.8 ± 1329	411.8 ± 402	0.2356

Abbreviations: ARDS, acute respiratory distress syndrome; ICU, intensive care unit; LOS, length of stay; AKI, acute kidney failure. ANOVA and Chi-Square test.

## Data Availability

The data supporting the findings of this study are included in this article. Further inquiries can be directed to the corresponding author.
